# Supplementary material for: Application of Wearable Sensors in Actuation and Control of Powered Ankle Exoskeletons: A Comprehensive Review
Source: Sensors (Basel). 2022 Mar 14;22(6):2244. doi: 10.3390/s22062244 (PMC8954890; doi:10.3390/s22062244)
Supplement: Supplementary file 1 [file sensors-22-02244-s001.zip › sensors-1595661-supplementary.pdf]

Scientific Method:

The selection process of the articles for review is illustrated in Figure S1. The articles published between January 2000 and June 2021 by PubMed, SAGE journals, IEEE Xplore, Scopus, ScienceDirect, Web of Science were searched for the following keywords: [‘ankle’+ ‘exoskeleton’] or [‘ankle’+ ‘robot’] or [‘ankle’ + ‘power’+ ‘orthosis’]. The search results were merged and after removing the duplicates, 5684 articles formed the initial selection pool. Then, the articles which did not include the word ‘ankle’ in the title were removed and 1020 articles remained in the pool. A review process was performed on the titles and abstracts of the remaining articles. The review papers as well as the articles concerning seated rehabilitation robots, unpowered passive devices and multi-joint assistive devices and suits, i.e., hip-knee-ankle and knee-ankle-foot exoskeletons were removed from the pool. Further, the articles concerning computer simulations only and / or Computer Aided Design (CAD) only that did not relate to any developed exoskeleton prototypes were also excluded from the list. This process brought the number of articles in the review list down to 172. The full papers of the remaining articles were then reviewed. The articles published by the same research group on the same PAE were grouped and reviewed together when extracting key information.

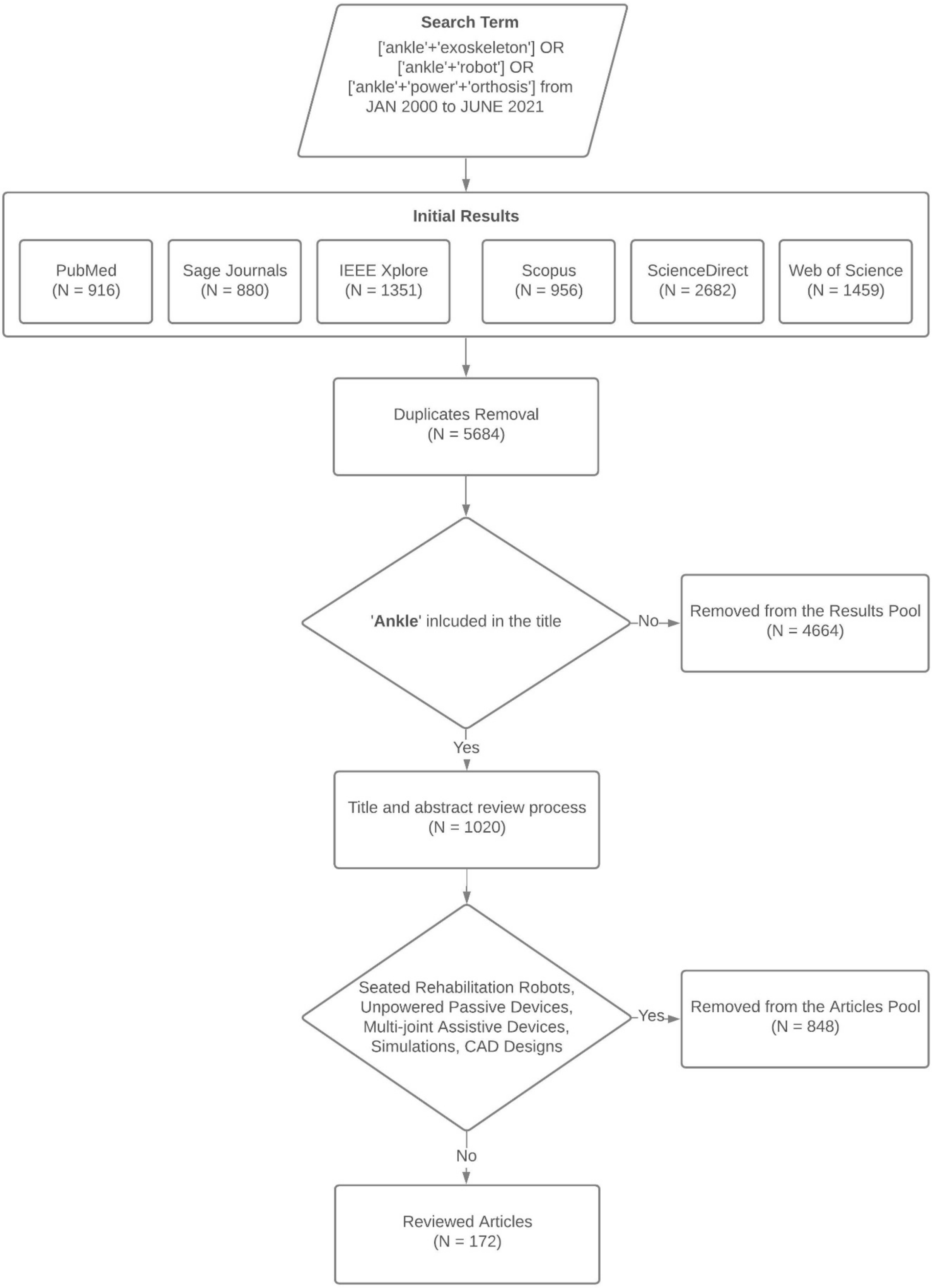

Figure S1. Schematic flow chart of the search and review process.

Table S1. General information extracted from the reviewed articles. Articles published by the same research group are presented with the same Research Group Code.

Research Group Code	References	Purpose	Bilateral	Degree of Freedom	Portability	Weight
**1**	[1]	Assistive Device	No	1 DOF Plantarflexion Only	Tethered	Not Specified
**2**	[2, 3]	General Augmentation	No	3 DOFs Dorsi/Plantarflexion, Eversion/Inversion, External/Internal Rotation	Tethered	0.77 kg
**3**	[4]	Assistive Device - Ankle Dyskinesia Patients and Functionally Degraded Elderly	No	1 DOF Dorsi/Plantarflexion	Portable	1.22 kg
**4**	[5]	General Augmentation and Assistive Device	Yes	1 DOF Dorsi/Plantarflexion	Portable	7.002 kg
**5**	[6]	Assistive Device	No	1 DOF Plantarflexion	Tethered	0.826 kg
	[7]	General Augmentation	Yes	1 DOF Plantarflexion	Tethered	0.415 kg
**6**	[8]	General Augmentation	Yes	1 DOF Plantarflexion	Tethered	Not Specified
**7**	[9-11]	Assistive Device – Drop Foot, Drag Foot and Hemiplegic Patients	No	1 DOF Dorsi/Plantarflexion	Tethered	2.8 kg
**8**	[12-14]	Assistive Device – Elderly	No	2 DOF Plantarflexion and Eversion/Inversion	Tethered	2.14 kg
**9**	[15]	Assistive Device	No	2 DOF Dorsi/Plantarflexion and Eversion/Inversion	Tethered	Not Specified
**10**	[16]	Assistive Device - Elderly and Gait-Impaired Patients	Yes	2 DOF Dorsi/Plantarflexion and Eversion/Inversion	Portable	0.869 kg
	[17]	Assistive Device - Hemiparetic Patients	Yes	2 DOF Dorsi/Plantarflexion and Eversion/Inversion	Portable	1.25 kg
	[18]	Assistive Device - Stroke Patients	Yes	2 DOF Dorsi/Plantarflexion and Eversion/Inversion	Portable	2.1 kg

**11**	[19]	Assistive Device -Stroke, Multiple Sclerosis, and Cerebral Palsy Patients	No	2 DOF Dorsi/Plantarflexion and Adduction/Abduction	Tethered	1.8 kg
**12**	[20-27]	Assistive Devices – Gait Impaired Patients	No	1 DOF Plantarflexion	Tethered	Alpha – 0.84 kg<br>Beta – 0.87 kg
**13**	[28, 29]	Assistive Device -<br>Stroke Patients and Locomotor disabled Patients	No	1 DOF Dorsi/Plantarflexion	Portable	2.8 kg
**14**	[30-39]	Assistive Device -Cerebral Palsy, Neuromuscular impaired, and Parkinson Patients	Yes	1 DOF Plantarflexion	Portable	Smaller Model- 1.85 kg<br>Larger Model- 2.20 kg
**15**	[40]	Assistive Device	Not Specified	Not Specified	Tethered	3.26 kg
	[41]	Assistive Device - Drop Foot Patients	No	1 DOF Dorsi/Plantarflexion	Tethered	1.323 kg
	[42]	Assistive Device - Drop Foot Patients	No	1 DOF Dorsiflexion	Portable	1.3 kg
**16**	[43]	Assistive Device - Stoke, and Cerebral Palsy Patients	No	1 DOF/ Plantarflexion	Tethered	2.5 kg
**17**	[44]	Assistive Device - Stroke Patients	No	1 DOF Dorsi/Plantarflexion	Portable	1.540 kg
	[45]	Assistive Device - Stroke Patients	Yes	2 DOF Dorsi/Plantarflexion and Eversion/Inversion	Portable	0.98 kg
**18**	[46-60]	Assistive Device	Yes (Interchangeable)	1 DOF Dorsi/Plantarflexion	Tethered	1.09 kg – 1.7 kg
	[61, 62]	Assistive Device	No	1 DOF Dorsi/Plantarflexion	Tethered	0.83 kg
	[63-67]	General Augmentation	Yes	1 DOF Plantarflexion	Tethered	1.52 kg
	[68]	Assistive Device - Stroke Patients	No	1 DOF Plantarflexion	Tethered	0.532 kg
	[69]	General Augmentation	Yes	1 DOF Plantarflexion	Tethered	2.08 kg

	[70]	Assistive Device	Yes	1 DOF Plantarflexion	Tethered	2.02 kg
	[71, 72]	General Augmentation	Yes	1 DOF Plantarflexion	Tethered	0.890 kg
**19**	[73]	Assistive Device - Patients with Chronic Ankle Instability	No	1 DOF Eversion/Inversion	Tethered	Not Specified
	[74]	Assistive Device	No	1 DOF Plantarflexion	Tethered	Not Specified
**20**	[75-80]	Assistive Device - Patients with Spinal Cord Injuries	Yes	1 DOF Dorsi/Plantarflexion	Portable	6.7 kg
**21**	[81, 82]	Assistive Device -Patients with Motor Injuries	No	1 DOF Dorsi/Plantarflexion	Portable	Not Specified
**22**	[83-88]	Assistive Device -Healthy young users	No	1 DOF Dorsi/Plantarflexion	Tethered	2.15 kg
**23**	[89]	Assistive Device	No	1 DOF Dorsiflexion	Tethered	Not Specified
**24**	[90]	General Augmentation	Yes	1 DOF Plantarflexion	Portable	3.97 kg
**25**	[91-93]	Assistive Device – Stroke and Neuromuscular Impaired Patients	No	1 DOF Dorsi/Plantarflexion	Tethered	1.04 kg
**26**	[94]	Assistive Device	No	1 DOF Dorsi/Plantarflexion	Tethered	Not Specified
	[95]	General Augmentation	No	1 DOF Plantarflexion	Tethered	Not Specified
**27**	[96]	Assistive Device – Drop Foot Patients	No	1 DOF Dorsi/Plantarflexion	Tethered	2.6 kg
	[97, 98]	General Augmentation	Yes	1 DOF Plantarflexion	Portable	3.6kg
	[99]	Assistive Device	Yes	1 DOF Plantarflexion	Portable	6.049 kg
**28**	[100]	Assistive Device - Stroke Patients	No	1 DOF Plantarflexion	Tethered	Not Specified
**29**	[101]	Assistive Device – Drop Foot Patients	No	1 DOF Dorsiflexion	Portable	1.2 kg
**30**	[102]	Assistive Device	No	1 DOF Plantarflexion	Portable	0.766 kg
	[103]	Assistive Device	No	1 DOF Plantarflexion	Tethered	1.35 kg
**31**	[104]	General Augmentation	Yes	1 DOF Plantarflexion	Portable	6.2 kg
**32**	[105-109]	Assistive Device – Drop Foot and Paretic Patients	No	1 DOF Dorsi/Plantarflexion	Portable	3.5 kg
**33**	[110]	General Augmentation	No	1 DOF Plantarflexion	Portable	2.045 kg
**34**	[111, 112]	Assistive Device –Stroke Patients	No	1 DOF Dorsiflexion	Portable	1 kg

**35**	[113]	Assistive Device – Mobility Impaired Patients	Yes	3 DOF Dorsi/Plantarflexion, Inversion/Eversion, Ad/Abduction	Tethered	2.6 kg
**36**	[114, 115]	Assistive Device	Yes	1 DOF Plantarflexion	Portable	10.1 kg
	[116-118]	General Augmentation	Yes	1 DOF Plantarflexion	Tethered	0.89 kg
	[119]	Assistive Device - Stroke Patients	No	1 DOF Dorsi/Plantarflexion	Tethered/ Portable	3.19 kg
	[120]	Assistive Device - Stroke Patients	No	1 DOF Dorsi/Plantarflexion	Portable	0.90 kg
	[121]	Assistive Device - Stroke Patients	No	1 DOF Dorsi/Plantarflexion	Portable	3.8 kg
	[122]	Assistive Device –Mobility Impaired Patients	No	1 DOF Dorsi/Plantarflexion	Tethered	0.90 kg
**37**	[123]	General Augmentation and Assistive Device - Patients with lower limb injuries	No	1 DOF Dorsi/Plantarflexion	Portable	3.4 kg
**38**	[124-128]	Assistive Device – Drop Foot and Stroke Patients	No	3 DOF Dorsi/Plantarflexion, Eversion/Inversion and Ad/Abduction	Tethered	3.6 kg
**39**	[129]	Assistive Device - Stroke Patients	No	1 DOF Dorsi/Plantarflexion	Portable	1.0 kg
**40**	[130-133]	General Augmentation and Assistive Device	Yes	1 DOF Plantarflexion	Tethered	2.08 kg
**41**	[134]	Assistive Device – Neuromuscular Impaired Patients	Yes	2 DOF Dorsi/Plantarflexion and Eversion/ Inversion	Portable	4.56 kg
	[135]	Assistive Device	Yes	1 DOF Dorsi/Plantarflexion	Tethered	5kg
**42**	[136-142]	Assistive Device -Multiple Sclerosis Patients	No	1 DOF Dorsi/Plantarflexion	Portable	3.1 kg
**43**	[143]	Assistive Device	No	1 DOF Dorsi/Plantarflexion	Tethered	3.0 kg
**44**	[144, 145]	Assistive Device	No	1 DOF Dorsi/Plantarflexion	Portable	1.2 kg
**45**	[146]	General Augmentation	No	1 DOF Dorsiflexion	Portable	2.2 kg
**47**	[147]	Assistive device -Paralysis Patients	No	1 DOF Dorsi/Plantarflexion	Tethered	Not Specified
**51**	[148-150]	Assistive Device	No	1 DOF Dorsi/Plantarflexion	Tethered	1.7 kg

**54**	[151]	Assistive Device – Drop Foot patients	No	1 DOF Dorsiflexion	Portable	2.0 kg
**56**	[152]	Assistive Device -Patients with existing Neurological issues	No	1 DOF Dorsi/Plantarflexion	Tethered	Not Specified
**57**	[153, 154]	Assistive Device -Neuromuscular Impaired Patients	No	2 DOF Dorsi/Plantarflexion and Eversion/Inversion	Portable	0.950 kg
**58**	[155]	Assistive Device - Neuromuscular Impaired Patients	No	2 DOF Dorsi/Plantarflexion and Eversion/Inversion	Tethered	1.1 kg
**61**	[156]	Assistive Device - Stroke Patients	No	1 DOF Dorsi/Plantarflexion	Not Specified	0.85 kg
**62**	[157]	Assistive Device - Individuals with spinal cord injuries and Stroke Patients	No	1 DOF Dorsi/Plantarflexion	Tethered	0.92 kg
**63**	[158, 159]	Assistive Device - Individuals with Peripheral or Central nervous system disorders	No	1 DOF Dorsi/Plantarflexion	Portable	Not Specified
**64**	[160, 161]	Assistive Device - Stroke Patients	No	2 DOF Dorsi/Plantarflexion and Eversion/ Inversion	Portable	Not Specified
**65**	[162, 163]	Assistive Device - Stroke Patients	No	1 DOF Plantarflexion	Tethered	0.95 kg
	[164]	Assistive Device	No	1 DOF Dorsi/Plantarflexion	Tethered	1.75 kg
	[165, 166]	Assistive Device - Stroke Patients	No	1 DOF Dorsi/Plantarflexion	Tethered	Not Specified
**66**	[167, 168]	Assistive Device	No	1 DOF Dorsi/Plantarflexion	Tethered	Not Specified
**69**	[169]	Assistive Device	No	1 DOF Dorsi/Plantarflexion	Tethered	Not Specified
**70**	[170, 171]	Assistive Device - Drop Foot Patients	No	1 DOF	Tethered	0.20 kg
**71**	[172]	General Augmentation	Yes	1 DOF Plantarflexion	Tethered	1.6 kg

References

1. Zhao, J., T. Yang, X. Sun, J. Dong, Z. Wang, and C. Yang, *Sliding mode control combined with extended state observer for an ankle exoskeleton driven by electrical motor.* Mechatronics, 2021. **76**: p. 102554. DOI: 10.1016/j.mechatronics.2021.102554.
2. Shao, Y., W. Zhang, K. Xu, and X. Ding, *Design of a Novel Compact Adaptive Ankle Exoskeleton for Walking Assistance*. 2019, Springer International Publishing. p. 2159-2168. DOI: 10.1007/978-3-030-20131-9_214.
3. Shao, Y., W. Zhang, Y. Su, and X. Ding, *Design and optimisation of load-adaptive actuator with variable stiffness for compact ankle exoskeleton.* Mechanism and Machine Theory, 2021. **161**: p. 104323. DOI: 10.1016/j.mechmachtheory.2021.104323.
4. Xing, K.W., Yinghan: Chen, Diansheng: Wang, Min: Lu, Sitong. *Flexible Ankle-Assisted Robot Technology Based on Rope Drive*. 2020. IEEE.DOI: 10.1109/rcar49640.2020.9303289.
5. Wang, T.M.P., X.: Hou, T. G.: Fan, Y. B.: Yang, X.: Herr, H. M.: Yang, X. B., *An untethered cable-driven ankle exoskeleton with plantarflexion-dorsiflexion bidirectional movement assistance.* Frontiers of Information Technology and Electronic Engineering, 2020. **21**(5): p. 723-739. DOI: 10.1631/FITEE.1900455.
6. Jackson, R.W.C., Steven H, *An experimental comparison of the relative benefits of work and torque assistance in ankle exoskeletons.* Journal of applied physiology, 2015. **119**(5): p. 541-557. https://journals.physiology.org/doi/pdf/10.1152/japplphysiol.01133.2014.
7. Nuckols, R.W.S., Gregory S., *Impact of elastic ankle exoskeleton stiffness on neuromechanics and energetics of human walking across multiple speeds.* Journal of NeuroEngineering and Rehabilitation, 2020. **17**(1). DOI: 10.1186/s12984-020-00703-4.
8. Shafer, B.A., S.A. Philius, R.W. Nuckols, J. McCall, A.J. Young, and G.S. Sawicki, *Neuromechanics and energetics of walking with an ankle exoskeleton using neuromuscular-model based control: a parameter study.* Frontiers in bioengineering and biotechnology, 2021. **9**.
9. Hwang, S., J. Kim, J. Yi, K. Tae, K. Ryu, and Y. Kim. *Development of an active ankle foot orthosis for the prevention of foot drop and toe drag*. in *2006 International Conference on Biomedical and Pharmaceutical Engineering*. 2006. IEEE.
10. Kim, J., S. Hwang, and Y. Kim. *Development of an active ankle-foot orthosis for hemiplegic patients*. in *Proceedings of the 1st international convention on Rehabilitation engineering & assistive technology: in conjunction with 1st Tan Tock Seng Hospital Neurorehabilitation Meeting*. 2007.
11. Kim, J., S. Hwang, R. Sohn, Y. Lee, and Y. Kim, *Development of an active ankle foot orthosis to prevent foot drop and toe drag in hemiplegic patients: A preliminary study.* Applied Bionics and Biomechanics, 2011. **8**(3, 4): p. 377-384.
12. Choi, H.S.L., Chang Hee: Baek, Yoon Su. *Design of a Pneumatic Actuated Ankle-Foot Orthosis which has Talocrural and Subtalar Joint*. 2019. IEEE.DOI: 10.1109/icorr.2019.8779486.

13. Choi, H.S.L., Chang Hee: Baek, Yoon Su, *Design and Validation of a Two-Degree-of-Freedom Powered Ankle-Foot Orthosis with Two Pneumatic Artificial Muscles.* Mechatronics, 2020. **72**: p. 102469. DOI: 10.1016/j.mechatronics.2020.102469.
14. Choi, H.S.B., Yoon Su, *Effects of the degree of freedom and assistance characteristics of powered ankle-foot orthoses on gait stability.* PLOS ONE, 2020. **15**(11): p. e0242000. DOI: 10.1371/journal.pone.0242000.
15. Lee, T., I. Kim, and Y.S. Baek, *Design of a 2DoF Ankle Exoskeleton with a Polycentric Structure and a Bi-Directional Tendon-Driven Actuator Controlled Using a PID Neural Network.* Actuators, 2021. **10**(1): p. 9. DOI: 10.3390/act10010009.
16. Choi, H.P., Young Jin: Seo, Keehong: Lee, Jusuk: Lee, Sang-Eui: Shim, Youngbo, *A Multifunctional Ankle Exoskeleton for Mobility Enhancement of Gait-Impaired Individuals and Seniors.* IEEE Robotics and Automation Letters, 2018. **3**(1): p. 411-418. DOI: 10.1109/lra.2017.2734239.
17. Seo, K.P., Young Jin: Lee, Jusuk: Hyung, Seungyong: Lee, Minhyung: Kim, Jeonghun: Choi, Hyundo: Shim, Youngbo. *RNN-Based On-Line Continuous Gait Phase Estimation from Shank-Mounted IMUs to Control Ankle Exoskeletons*. 2019. IEEE.DOI: 10.1109/icorr.2019.8779554.
18. Lee, M.K., Jeonghun: Hyung, Seungyong: Lee, Jusuk: Seo, Keehong: Park, Young Jin: Cho, Joonkee: Choi, Byung-Kwon: Shim, Youngbo: Choi, Hyundo, *A Compact Ankle Exoskeleton With a Multiaxis Parallel Linkage Mechanism.* IEEE/ASME Transactions on Mechatronics, 2021. **26**(1): p. 191-202. DOI: 10.1109/tmech.2020.3008372.
19. Kirtas, O.S., Yagiz: Bayraker, Melih: Baskaya, Faik: Basturk, Halil: Samur, Evren, *Design, implementation, and evaluation of a backstepping control algorithm for an active ankle–foot orthosis.* Control Engineering Practice, 2021. **106**: p. 104667. DOI: 10.1016/j.conengprac.2020.104667.
20. Witte, K.A., J. Zhang, R.W. Jackson, and S.H. Collins. *Design of two lightweight, high-bandwidth torque-controlled ankle exoskeletons*. in *2015 IEEE International Conference on Robotics and Automation (ICRA)*. 2015. IEEE.
21. Steele, K.M.J., Rachel W.: Shuman, Benjamin R.: Collins, Steven H., *Muscle recruitment and coordination with an ankle exoskeleton.* Journal of Biomechanics, 2017. **59**: p. 50-58. DOI: 10.1016/j.jbiomech.2017.05.010.
22. Jackson, R.W.C., Steven H., *Heuristic-Based Ankle Exoskeleton Control for Co-Adaptive Assistance of Human Locomotion.* IEEE Transactions on Neural Systems and Rehabilitation Engineering, 2019. **27**(10): p. 2059-2069. DOI: 10.1109/tnsre.2019.2936383.
23. Wang, W.C., Jianyu: Ji, Yandong: Jin, Wei: Liu, Jingtai: Zhang, Juanjuan, *Evaluation of Lower Leg Muscle Activities During Human Walking Assisted by an Ankle Exoskeleton.* IEEE Transactions on Industrial Informatics, 2020. **16**(11): p. 7168-7176. DOI: 10.1109/tii.2020.2974232.
24. Zhang, J., P. Fiers, K.A. Witte, R.W. Jackson, K.L. Poggensee, C.G. Atkeson, and S.H.J.S. Collins, *Human-in-the-loop optimization of exoskeleton assistance during walking.* 2017. **356**(6344): p. 1280-1284.

25. Witte, K.A.F., Pieter: Sheets-Singer, Alison L.: Collins, Steven H., *Improving the energy economy of human running with powered and unpowered ankle exoskeleton assistance.* Science Robotics, 2020. **5**(40): p. eaay9108. DOI: 10.1126/scirobotics.aay9108.
26. Han, H., W. Wang, F. Zhang, X. Li, J. Chen, J. Han, and J. Zhang, *Selection of Muscle-Activity-Based Cost Function in Human-in-the-Loop Optimization of Multi-Gait Ankle Exoskeleton Assistance.* IEEE Transactions on Neural Systems and Rehabilitation Engineering, 2021. **29**: p. 944-952. DOI: 10.1109/tnsre.2021.3082198.
27. Zhang, J.C., Chien Chern: Collins, Steven H. *Experimental comparison of torque control methods on an ankle exoskeleton during human walking*. 2015. IEEE.DOI: 10.1109/icra.2015.7139980.
28. Manchola, M., D. Serrano, D. Gómez, F. Ballen, D. Casas, M. Munera, and C.A. Cifuentes. *T-FLEX: Variable stiffness ankle-foot orthosis for gait assistance*. in *International Symposium on Wearable Robotics*. 2018. Springer.
29. Gomez-Vargas, D., F. Ballen-Moreno, P. Barria, R. Aguilar, J.M. Azorín, M. Munera, and C.A. Cifuentes, *The Actuation System of the Ankle Exoskeleton T-FLEX: First Use Experimental Validation in People with Stroke.* Brain Sciences, 2021. **11**(4): p. 412. DOI: 10.3390/brainsci11040412.
30. Lerner, Z.F.G., Gian Maria: Bair, Michael O.: Lawson, Jenny L.: Luque, Jason: Harvey, Taryn A.: Lerner, Andrea T., *An Untethered Ankle Exoskeleton Improves Walking Economy in a Pilot Study of Individuals With Cerebral Palsy.* IEEE Transactions on Neural Systems and Rehabilitation Engineering, 2018. **26**(10): p. 1985-1993. DOI: 10.1109/tnsre.2018.2870756.
31. Gasparri, G.M.B., Michael Owen: Libby, Robert Patrick: Lerner, Zachary Forest. *Verification of a Robotic Ankle Exoskeleton Control Scheme for Gait Assistance in Individuals with Cerebral Palsy*. 2018. IEEE.DOI: 10.1109/iros.2018.8593904.
32. Lerner, Z.F.H., Taryn A.: Lawson, Jennifer L., *A Battery-Powered Ankle Exoskeleton Improves Gait Mechanics in a Feasibility Study of Individuals with Cerebral Palsy.* Annals of Biomedical Engineering, 2019. **47**(6): p. 1345-1356. DOI: 10.1007/s10439-019-02237-w.
33. Lerner, Z.F.C., Benjamin C.: Remec, Nushka M. *Adaptation of Gait Energetics to Ankle Exoskeleton Assistance Within and Across Visits: A Clinical Case Series*. 2019. IEEE.DOI: 10.1109/wearracon.2019.8719627.
34. Gasparri, G.M.L., Jason: Lerner, Zachary F., *Proportional Joint-Moment Control for Instantaneously Adaptive Ankle Exoskeleton Assistance.* IEEE Transactions on Neural Systems and Rehabilitation Engineering, 2019. **27**(4): p. 751-759. DOI: 10.1109/tnsre.2019.2905979.
35. Orekhov, G.F., Ying: Luque, Jason: Lerner, Zachary F., *Ankle Exoskeleton Assistance Can Improve Over-Ground Walking Economy in Individuals With Cerebral Palsy.* IEEE Transactions on Neural Systems and Rehabilitation Engineering, 2020. **28**(2): p. 461-467. DOI: 10.1109/tnsre.2020.2965029.
36. Jain, T.K.C., Valerie: Lerner, Zachary F., *Effects of Lightweight Wearable Ankle Exoskeleton in an Individual With Parkinson Disease A Case Report.* Topics in Geriatric Rehabilitation, 2020. **36**(3): p. 146-151. DOI: 10.1097/tgr.0000000000000273.

37. Fang, Y.O., Greg: Lerner, Zachary F., *Adaptive ankle exoskeleton gait training demonstrates acute neuromuscular and spatiotemporal benefits for individuals with cerebral palsy: A pilot study*. Gait & Posture, 2020. DOI: 10.1016/j.gaitpost.2020.11.005.
38. Conner, B.C.L., Jason: Lerner, Zachary F., *Adaptive Ankle Resistance from a Wearable Robotic Device to Improve Muscle Recruitment in Cerebral Palsy.* Annals of Biomedical Engineering, 2020. **48**(4): p. 1309-1321. DOI: 10.1007/s10439-020-02454-8.
39. Fang, Y. and Z.F. Lerner, *Feasibility of Augmenting Ankle Exoskeleton Walking Performance With Step Length Biofeedback in Individuals With Cerebral Palsy.* IEEE Transactions on Neural Systems and Rehabilitation Engineering, 2021. **29**: p. 442-449. DOI: 10.1109/tnsre.2021.3055796.
40. Lv, G., H. Zhu, T. Elery, L. Li, and R.D. Gregg. *Experimental implementation of underactuated potential energy shaping on a powered ankle-foot orthosis*. in *2016 IEEE International Conference on Robotics and Automation (ICRA)*. 2016. IEEE.
41. Kumar, S.Z., Matthew: Bolivar-Nieto, Edgar: Gregg, Robert D.: Gans, Nicholas, *Extremum Seeking Control for Stiffness Auto-Tuning of a Quasi-Passive Ankle Exoskeleton.* IEEE Robotics and Automation Letters, 2020: p. 1-1. DOI: 10.1109/lra.2020.3001541.
42. Allen, D.P., R. Little, J. Laube, J. Warren, W. Voit, and R.D. Gregg, *Towards an ankle-foot orthosis powered by a dielectric elastomer actuator.* Mechatronics, 2021. **76**: p. 102551. DOI: 10.1016/j.mechatronics.2021.102551.
43. Zhou, Y.L., Lu. *Development and Testing of a User-adaptive Ankle Foot Orthosis*. 2020. IEEE.DOI: 10.1109/icarm49381.2020.9195386.
44. Kwon, J.P., Ji-Hong: Ku, Subyeong: Jeong, Yeonghyeon: Paik, Nam-Jong: Park, Yong-Lae, *A Soft Wearable Robotic Ankle-Foot-Orthosis for Post-Stroke Patients.* IEEE Robotics and Automation Letters, 2019. **4**(3): p. 2547-2552. DOI: 10.1109/lra.2019.2908491.
45. Xia, H.K., Junghan: Pathak, Prabhat: Ahn, Jooeun: Shull, Peter B.: Park, Yong-Lae. *Design of A Multi-Functional Soft Ankle Exoskeleton for Foot-Drop Prevention, Propulsion Assistance, and Inversion/Eversion Stabilization*. 2020. IEEE.DOI: 10.1109/biorob49111.2020.9224420.
46. Ferris, D.P.C., J. M.: Hannaford, B., *An ankle-foot orthosis powered by artificial pneumatic muscles.* Journal of Applied Biomechanics, 2005. **21**(2): p. 189-197. DOI: 10.1123/jab.21.2.189.
47. Sawicki, G.S., K.E. Gordon, and D.P. Ferris. *Powered Lower Limb Orthoses: Applications in Motor Adaptation and Rehabilitation*. 2005. IEEE.DOI: 10.1109/icorr.2005.1501086.
48. Sawicki, G.S.D., Antoinette: Ferris, Daniel P., *The effects of powered ankle-foot orthoses on joint kinematics and muscle activation during walking in individuals with incomplete spinal cord injury.* Journal of NeuroEngineering and Rehabilitation, 2006. **3**(1): p. 3. DOI: 10.1186/1743-0003-3-3.
49. Gordon, K.E.S., Gregory S.: Ferris, Daniel P., *Mechanical performance of artificial pneumatic muscles to power an ankle–foot orthosis.* Journal of Biomechanics, 2006. **39**(10): p. 1832-1841. DOI: 10.1016/j.jbiomech.2005.05.018.

50. Ferris, D.P.G., Keith E.: Sawicki, Gregory S.: Peethambaran, Ammanath, *An improved powered ankle–foot orthosis using proportional myoelectric control.* Gait & Posture, 2006. **23**(4): p. 425-428. DOI: 10.1016/j.gaitpost.2005.05.004.
51. Gordon, K.E.F., Daniel P., *Learning to walk with a robotic ankle exoskeleton.* Journal of Biomechanics, 2007. **40**(12): p. 2636-2644. DOI: 10.1016/j.jbiomech.2006.12.006.
52. Cain, S.M.G., Keith E.: Ferris, Daniel P., *Locomotor adaptation to a powered ankle-foot orthosis depends on control method.* Journal of Neuroengineering and Rehabilitation, 2007. **4**. DOI: 10.1186/1743-0003-4-48.
53. Sawicki, G.S.F., D. P., *Mechanics and energetics of level walking with powered ankle exoskeletons.* Journal of Experimental Biology, 2008. **211**(9): p. 1402-1413. DOI: 10.1242/jeb.009241.
54. Sawicki, G.S.F., D. P., *Powered ankle exoskeletons reveal the metabolic cost of plantar flexor mechanical work during walking with longer steps at constant step frequency.* Journal of Experimental Biology, 2009. **212**(1): p. 21-31. DOI: 10.1242/jeb.017269.
55. Sawicki, G.S.F., D. P., *Mechanics and energetics of incline walking with robotic ankle exoskeletons.* Journal of Experimental Biology, 2009. **212**(1): p. 32-41. DOI: 10.1242/jeb.017277.
56. Kinnaird, C.R.F., D. P., *Medial Gastrocnemius Myoelectric Control of a Robotic Ankle Exoskeleton.* IEEE Transactions on Neural Systems and Rehabilitation Engineering, 2009. **17**(1): p. 31-37. DOI: 10.1109/tnsre.2008.2008285.
57. Kao, P.-C. and D.P. Ferris, *Motor adaptation during dorsiflexion-assisted walking with a powered orthosis.* Gait & Posture, 2009. **29**(2): p. 230-236. DOI: 10.1016/j.gaitpost.2008.08.014.
58. Kao, P.-C.L., Cara L.: Ferris, Daniel P., *Short-term locomotor adaptation to a robotic ankle exoskeleton does not alter soleus Hoffmann reflex amplitude.* Journal of NeuroEngineering and Rehabilitation, 2010. **7**(1): p. 33. DOI: 10.1186/1743-0003-7-33.
59. Kao, P.-C.L., Cara L.: Ferris, Daniel P., *Joint kinetic response during unexpectedly reduced plantar flexor torque provided by a robotic ankle exoskeleton during walking.* Journal of Biomechanics, 2010. **43**(7): p. 1401-1407. DOI: 10.1016/j.jbiomech.2009.12.024.
60. Kao, P.-C.L., Cara L.: Ferris, Daniel P., *Invariant ankle moment patterns when walking with and without a robotic ankle exoskeleton.* Journal of Biomechanics, 2010. **43**(2): p. 203-209. DOI: 10.1016/j.jbiomech.2009.09.030.
61. Malcolm, P.S., V.: Van Caekenberghe, I.: De Clercq, D., *Experimental study of the influence of the m. tibialis anterior on the walk-to-run transition by means of a powered ankle-foot exoskeleton.* Gait & Posture, 2009. **29**(1): p. 6-10. DOI: 10.1016/j.gaitpost.2008.05.016.
62. Malcolm, P.F., P.: Segers, V.: Van Caekenberghe, I.: Lenoir, M.: De Clercq, D., *Experimental study on the role of the ankle push off in the walk-to-run transition by means of a powered ankle-foot-exoskeleton.* Gait & Posture, 2009. **30**(3): p. 322-327. DOI: 10.1016/j.gaitpost.2009.06.002.
63. Malcolm, P., W. Derave, S. Galle, and D. De Clercq, *A Simple Exoskeleton That Assists Plantarflexion Can Reduce the Metabolic Cost of Human Walking.* PLoS ONE, 2013. **8**(2): p. e56137. DOI: 10.1371/journal.pone.0056137.

64. Galle, S.M., P.: Derave, W.: De Clercq, D., *Adaptation to walking with an exoskeleton that assists ankle extension.* Gait & Posture, 2013. **38**(3): p. 495-499. DOI: 10.1016/j.gaitpost.2013.01.029.
65. Galle, S.M., P.: Derave, W.: De Clercq, D., *Enhancing performance during inclined loaded walking with a powered ankle–foot exoskeleton.* European Journal of Applied Physiology, 2014. **114**(11): p. 2341-2351. DOI: 10.1007/s00421-014-2955-1.
66. Galle, S., P. Malcolm, W. Derave, and D. De Clercq, *Uphill walking with a simple exoskeleton: Plantarflexion assistance leads to proximal adaptations.* Gait & Posture, 2015. **41**(1): p. 246-251. DOI: 10.1016/j.gaitpost.2014.10.015.
67. Galle, S., W. Derave, F. Bossuyt, P. Calders, P. Malcolm, and D. De Clercq, *Exoskeleton plantarflexion assistance for elderly.* Gait & Posture, 2017. **52**: p. 183-188. DOI: 10.1016/j.gaitpost.2016.11.040.
68. Takahashi, K.Z.L., Michael D.: Sawicki, Gregory S., *A neuromechanics-based powered ankle exoskeleton to assist walking post-stroke: a feasibility study.* Journal of NeuroEngineering and Rehabilitation, 2015. **12**(1): p. 23. DOI: 10.1186/s12984-015-0015-7.
69. Koller, J.R.J., Daniel A.: Ferris, Daniel P.: Remy, C. David, *Learning to walk with an adaptive gain proportional myoelectric controller for a robotic ankle exoskeleton.* Journal of NeuroEngineering and Rehabilitation, 2015. **12**(1). DOI: 10.1186/s12984-015-0086-5.
70. Koller, J.R., D.H. Gates, D.P. Ferris, and C.D. Remy. *'Body-in-the-Loop'Optimization of Assistive Robotic Devices: A Validation Study*. in *Robotics: Science and Systems*. 2016.
71. Galle, S., P. Malcolm, S.H. Collins, and D. De Clercq, *Reducing the metabolic cost of walking with an ankle exoskeleton: interaction between actuation timing and power.* Journal of NeuroEngineering and Rehabilitation, 2017. **14**(1). DOI: 10.1186/s12984-017-0235-0.
72. Antonellis, P.G., Samuel: De Clercq, Dirk: Malcolm, Philippe, *Altering gait variability with an ankle exoskeleton.* PLOS ONE, 2018. **13**(10): p. e0205088. DOI: 10.1371/journal.pone.0205088.
73. Thalman, C.M.L., Hyunglae. *Design and Validation of a Soft Robotic Ankle-Foot Orthosis (SR-AFO) Exosuit for Inversion and Eversion Ankle Support*. 2020. IEEE.DOI: 10.1109/icra40945.2020.9197531.
74. Thalman, C.M.H., Tiffany: Lee, Hyunglae. *Toward A Soft Robotic Ankle-Foot Orthosis (SR-AFO) Exosuit for Human Locomotion: Preliminary Results in Late Stance Plantarflexion Assistance*. 2020. IEEE.DOI: 10.1109/robosoft48309.2020.9116050.
75. Meijneke, C.V.D., Wietse: Van Der Kooij, Herman. *Achilles: An autonomous lightweight ankle exoskeleton to provide push-off power*. 2014. IEEE.DOI: 10.1109/biorob.2014.6913898.
76. Van Dijk, W.M., Cory: Van Der Kooij, Herman, *Evaluation of the Achilles Ankle Exoskeleton.* IEEE Transactions on Neural Systems and Rehabilitation Engineering, 2017. **25**(2): p. 151-160. DOI: 10.1109/tnsre.2016.2527780.
77. Emmens, A.R.V.A., Edwin H. F.: Van Der Kooij, Herman, *Effects of a powered ankle-foot orthosis on perturbed standing balance.* Journal of NeuroEngineering and Rehabilitation, 2018. **15**(1). DOI: 10.1186/s12984-018-0393-8.

78. Tamburella, F.T., N. L.: Pisotta, I.: Masciullo, M.: Arquilla, M.: van Asseldonk, E. H. F.: van der Kooij, H.: Wu, A. R.: Dzeladini, F.: Ijspeert, A. J.: Molinari, M., *Neuromuscular Controller Embedded in a Powered Ankle Exoskeleton: Effects on Gait, Clinical Features and Subjective Perspective of Incomplete Spinal Cord Injured Subjects.* IEEE Trans Neural Syst Rehabil Eng, 2020. **28**(5): p. 1157-1167. DOI: 10.1109/tnsre.2020.2984790.
79. Tamburella, F.T., N. L.: Masciullo, M.: Pisotta, I.: Arquilla, M.: van Asseldonk, E. H. F.: van der Kooij, H.: Wu, A. R.: Dzeladini, F.: Ijspeert, A. J.: Molinari, M., *Gait training with Achilles ankle exoskeleton in chronic incomplete spinal cord injury subjects.* Journal of biological regulators and homeostatic agents, 2020. **34**(5 Suppl. 3): p. 147-Medicine. <Go to ISI>://MEDLINE:33386045.
80. Dzeladini, F.W., Amy R.: Renjewski, Daniel: Arami, Arash: Burdet, Etienne: Van Asseldonk, Edwin: Van Der Kooij, Herman: Ijspeert, Auke J. *Effects of a neuromuscular controller on a powered ankle exoskeleton during human walking*. 2016. IEEE.DOI: 10.1109/biorob.2016.7523694.
81. Pinheiro, C.F., Joana: Magalhães, Nuno: Santos, Cristina P., *Wearable Biofeedback Improves Human-Robot Compliance during Ankle-Foot Exoskeleton-Assisted Gait Training: A Pre-Post Controlled Study in Healthy Participants.* Sensors, 2020. **20**(20): p. 5876. DOI: 10.3390/s20205876.
82. Lopes, J.P., Cristiana: Figueiredo, Joana: Reis, Luis P.: Santos, Cristina P. *Assist-as-needed Impedance Control Strategy for a Wearable Ankle Robotic Orthosis*. 2020. IEEE.DOI: 10.1109/icarsc49921.2020.9096186.
83. Moltedo, M.B., Tomislav: Junius, Karen: Vanderborght, Bram: Lefeber, Dirk. *Mechanical design of a lightweight compliant and adaptable active ankle foot orthosis*. 2016. IEEE.DOI: 10.1109/biorob.2016.7523798.
84. Moltedo, M.B., Tomislav: Langlois, Kevin: Junius, Karen: Vanderborght, Bram: Lefeber, Dirk. *Design and experimental evaluation of a lightweight, high-torque and compliant actuator for an active ankle foot orthosis*. 2017. IEEE.DOI: 10.1109/icorr.2017.8009260.
85. Moltedo, M.B., Tomislav: Langlois, Kevin: Junius, Karen: Vanderborght, Bram: Lefeber, Dirk, *A Compliant Lightweight and Adaptable Active Ankle Foot Orthosis for Robotic Rehabilitation*. 2017, Springer International Publishing. p. 45-49. DOI: 10.1007/978-3-319-46532-6_8.
86. Langlois, K.M., Marta: Bacek, Tomislav: Rodriguez-Guerrero, Carlos: Vanderborght, Bram: Lefeber, Dirk. *Design and Development of Customized Physical Interfaces to Reduce Relative Motion Between the User and a Powered Ankle Foot Exoskeleton*. 2018. IEEE.DOI: 10.1109/biorob.2018.8487706.
87. Moltedo, M.C., Gaia: Baček, Tomislav: Lataire, John: Vanderborght, Bram: Lefeber, Dirk: Rodriguez-Guerrero, Carlos, *Variable stiffness ankle actuator for use in robotic-assisted walking: Control strategy and experimental characterization.* Mechanism and Machine Theory, 2019. **134**: p. 604-624. DOI: 10.1016/j.mechmachtheory.2019.01.017.

88. Moltedo, M.B., Tomislav: Serrien, Ben: Langlois, Kevin: Vanderborght, Bram: Lefeber, Dirk: Rodriguez-Guerrero, Carlos, *Walking with a powered ankle-foot orthosis: the effects of actuation timing and stiffness level on healthy users.* Journal of NeuroEngineering and Rehabilitation, 2020. **17**(1). DOI: 10.1186/s12984-020-00723-0.
89. Kamarudin, N.E.M.N., I. N. A. M.: Faudzi, A. A. M.: Khamis, N.: Razif, M. R. M.: Zulkarnain, N.: Abidin, A. F. Z., *Soft actuated foot exoskeleton for ankle rehabilitation.* International Journal of Advanced Trends in Computer Science and Engineering, 2020. **9**(1.4 Special Issue): p. 367-373. DOI: 10.30534/ijatcse/2020/5391.42020.
90. Ingraham, K.A.R., C. David: Rouse, Elliott J. *User preference of applied torque characteristics for bilateral powered ankle exoskeletons*. 2020. IEEE.DOI: 10.1109/biorob49111.2020.9224358.
91. Zhang, Y.K., Roger J.: Nolan, Karen J.: Zanotto, Damiano. *Design and Evaluation of an Active/Semiactive Ankle-Foot Orthosis for Gait Training*. 2018. IEEE.DOI: 10.1109/biorob.2018.8487973.
92. Zhang, Y.N., Karen J.: Zanotto, Damiano, *Oscillator-Based Transparent Control of an Active/Semiactive Ankle-Foot Orthosis.* IEEE Robotics and Automation Letters, 2019. **4**(2): p. 247-253. DOI: 10.1109/lra.2018.2886400.
93. Zhang, Y.K., Roger J.: Nolan, Karen J.: Zanotto, Damiano, *Preliminary Validation of a Cable-Driven Powered Ankle–Foot Orthosis With Dual Actuation Mode.* IEEE Transactions on Medical Robotics and Bionics, 2019. **1**(1): p. 30-37. DOI: 10.1109/tmrb.2019.2895787.
94. Li, M.P., Muye: Xiang, Kui: Ju, Zhaojie: Zhou, Shenpei. *Design and Reflex Control for a Series Elastic Actuator Based Ankle Joint Emulator*. 2018. IEEE.DOI: 10.1109/icarm.2018.8610713.
95. Yan, H.T., Biwei: Xiang, Kui: Pang, Muye. *Human-in-the-Loop Optimization Control for the Ankle Exoskeleton During Walking Based on Iterative Learning and Particle Swarm Optimization Algorithm*. 2019. IEEE.DOI: 10.1109/icarm.2019.8834170.
96. Blaya, J.A.H., H., *Adaptive Control of a Variable-Impedance Ankle-Foot Orthosis to Assist Drop-Foot Gait.* IEEE Transactions on Neural Systems and Rehabilitation Engineering, 2004. **12**(1): p. 24-31. DOI: 10.1109/tnsre.2003.823266.
97. Mooney, L.M., E.J. Rouse, and H.M. Herr, *Autonomous exoskeleton reduces metabolic cost of human walking during load carriage.* Journal of NeuroEngineering and Rehabilitation, 2014. **11**(1): p. 80. DOI: 10.1186/1743-0003-11-80.
98. Mooney, L.M. and H.M. Herr, *Biomechanical walking mechanisms underlying the metabolic reduction caused by an autonomous exoskeleton.* Journal of NeuroEngineering and Rehabilitation, 2016. **13**(1). DOI: 10.1186/s12984-016-0111-3.
99. Wu, A.Y., Xingbang: Kuan, Jiun-Yih: Herr, Hugh M. *An Autonomous Exoskeleton for Ankle Plantarflexion Assistance*. 2019. IEEE.DOI: 10.1109/icra.2019.8793913.
100. McCain, E.M.D., Taylor J. M.: Giest, Tracy N.: Nuckols, Richard W.: Lewek, Michael D.: Saul, Katherine R.: Sawicki, Gregory S., *Mechanics and energetics of post-stroke walking aided by a powered ankle exoskeleton with speed-adaptive myoelectric control.* Journal of NeuroEngineering and Rehabilitation, 2019. **16**(1). DOI: 10.1186/s12984-019-0523-y.

101. Martinez-Hernandez, U.R.-S., Adrian: Cedeno-Campos, Victor: Dehghani-Sanij, Abbas A. *Towards an intelligent wearable ankle robot for assistance to foot drop*. 2019. IEEE.DOI: 10.1109/smc.2019.8914170.
102. Liu, J.X., Caihua: Fu, Chenglong, *An Ankle Exoskeleton Using a Lightweight Motor to Create High Power Assistance for Push-Off.* Journal of Mechanisms and Robotics-Transactions of the Asme, 2019. **11**(4). DOI: 10.1115/1.4043456.
103. Dong, W.L., Chongchong: Zhang, Qin: Xiong, Caihua. *Design and Evaluation of an Active Ankle Exoskeleton in Gait Assistance*. 2019. IEEE.DOI: 10.1109/aim.2019.8868740.
104. Khazoom, C.V., Catherine: Bigue, Jean-Philippe Lucking: Grenier, Jordane: Girard, Alexandre: Plante, Jean-Sebastien, *Design and Control of a Multifunctional Ankle Exoskeleton Powered by Magnetorheological Actuators to Assist Walking, Jumping, and Landing.* IEEE Robotics and Automation Letters, 2019. **4**(3): p. 3083-3090. DOI: 10.1109/lra.2019.2924852.
105. Arnez-Paniagua, V.R., Hala: Mohammed, Samer: Amirat, Yacine, *Adaptive Control of an Actuated Ankle Foot Orthosis for Foot-Drop Correction.* IFAC-PapersOnLine, 2017. **50**(1): p. 1384-1389. DOI: 10.1016/j.ifacol.2017.08.237.
106. Guerrero-Castellanos, J.F.R., H.: Arnez-Paniagua, V.: Linares-Flores, J.: Saynes-Torres, L.: Mohammed, S., *Robust Active Disturbance Rejection Control via Control Lyapunov Functions: Application to Actuated-Ankle–Foot-Orthosis.* Control Engineering Practice, 2018. **80**: p. 49-60. DOI: 10.1016/j.conengprac.2018.08.008.
107. Arnez-Paniagua, V.R., H.: Amirat, Y.: Mohammed, S.: Ghedira, M.: Gracies, J. M. *Modified Adaptive Control of an Actuated Ankle Foot Orthosis to assist Paretic Patients*. 2018. IEEE.DOI: 10.1109/iros.2018.8594046.
108. Huo, W.A.-P., Victor: Ding, Guangzheng: Amirat, Yacine: Mohammed, Samer, *Adaptive Proxy-Based Controller of an Active Ankle Foot Orthosis to Assist Lower Limb Movements of Paretic Patients.* Robotica, 2019. **37**(12): p. 2147-2164. DOI: 10.1017/s0263574719000250.
109. Arnez-Paniagua, V.R., H.: Amirat, Y.: Ghedira, M.: Gracies, J. M.: Mohammed, S., *Adaptive control of an actuated ankle foot orthosis for paretic patients.* Control Engineering Practice, 2019. **90**: p. 207-220. DOI: 10.1016/j.conengprac.2019.06.003.
110. Bougrinat, Y.A., Sofiane: Raison, Maxime, *Design and development of a lightweight ankle exoskeleton for human walking augmentation.* Mechatronics, 2019. **64**: p. 102297. DOI: 10.1016/j.mechatronics.2019.102297.
111. Yeung, L.-F.O., Corinna: Pang, Man-Kit: Wai, Hon-Wah: Soo, Oi-Yan: Li, Sheung-Wai: Tong, Kai-Yu. *Design of an exoskeleton ankle robot for robot-assisted gait training of stroke patients*. 2017. IEEE.DOI: 10.1109/icorr.2017.8009248.
112. Yeung, L.-F.O., Corinna: Pang, Man-Kit: Wai, Hon-Wah: Soo, Oi-Yan: Li, Sheung-Wai: Tong, Kai-Yu, *Randomized controlled trial of robot-assisted gait training with dorsiflexion assistance on chronic stroke patients wearing ankle-foot-orthosis.* Journal of NeuroEngineering and Rehabilitation, 2018. **15**(1). DOI: 10.1186/s12984-018-0394-7.
113. Weerasingha, A.H.P., A. D. K. H.: Withanage, W. P. K.: Ranaweera, R. K. P. S.: Gopura, R. A. R. C. *C-JAE: 3 DOF Robotic Ankle Exoskeleton with Compatible Joint Axes*. 2018. IEEE.DOI: 10.1109/mercon.2018.8422004.

114. Asbeck, A.T., S.M.M. De Rossi, K.G. Holt, and C.J. Walsh, *A biologically inspired soft exosuit for walking assistance.* The International Journal of Robotics Research, 2015. **34**(6): p. 744-762. DOI: 10.1177/0278364914562476.
115. Lee, S., S. Crea, P. Malcolm, I. Galiana, A. Asbeck, and C. Walsh. *Controlling negative and positive power at the ankle with a soft exosuit*. 2016. IEEE.DOI: 10.1109/icra.2016.7487531.
116. Quinlivan, B., S. Lee, P. Malcolm, D. Rossi, M. Grimmer, C. Siviy, N. Karavas, D. Wagner, A. Asbeck, and I. Galiana, *Assistance magnitude versus metabolic cost reductions for a tethered multiarticular soft exosuit.* Science robotics, 2017. **2**(2): p. eaah4416.
117. Malcolm, P.L., Sangjun: Crea, Simona: Siviy, Christopher: Saucedo, Fabricio: Galiana, Ignacio: Panizzolo, Fausto A.: Holt, Kenneth G.: Walsh, Conor J., *Varying negative work assistance at the ankle with a soft exosuit during loaded walking.* Journal of NeuroEngineering and Rehabilitation, 2017. **14**(1). DOI: 10.1186/s12984-017-0267-5.
118. Grimmer, M.Q., Brendan T.: Lee, Sangjun: Malcolm, Philippe: Rossi, Denise Martineli: Siviy, Christopher: Walsh, Conor J., *Comparison of the human-exosuit interaction using ankle moment and ankle positive power inspired walking assistance.* Journal of Biomechanics, 2019. **83**: p. 76-84. DOI: 10.1016/j.jbiomech.2018.11.023.
119. Awad, L.N.B., Jaehyun: O'Donnell, Kathleen: De Rossi, Stefano M. M.: Hendron, Kathryn: Sloot, Lizeth H.: Kudzia, Pawel: Allen, Stephen: Holt, Kenneth G.: Ellis, Terry D.: Walsh, Conor J., *A soft robotic exosuit improves walking in patients after stroke.* Science Translational Medicine, 2017. **9**(400): p. eaai9084. DOI: 10.1126/scitranslmed.aai9084.
120. Sloot, L.B., J.: Baker, L.: O'Donnell, K.: Menard, N.: Porciuncula, F.: Choe, D.: Ellis, T.: Awad, L.: Walsh, C., *O 089 - A soft robotic exosuit assisting the paretic ankle in patients post-stroke: Effect on muscle activation during overground walking.* Gait & Posture, 2018. DOI: https://doi.org/10.1016/j.gaitpost.2018.06.124.
121. Bae, J.S., Christopher: Rouleau, Michael: Menard, Nicolas: Odonnell, Kathleen: Geliana, Ignacio: Athanassiu, Maria: Ryan, Danielle: Bibeau, Christine: Sloot, Lizeth: Kudzia, Pawel: Ellis, Terry: Awad, Louis: Walsh, Conor J. *A Lightweight and Efficient Portable Soft Exosuit for Paretic Ankle Assistance in Walking After Stroke*. 2018. IEEE.DOI: 10.1109/icra.2018.8461046.
122. Bae, J., L.N. Awad, A. Long, K. O'Donnell, K. Hendron, K.G. Holt, T.D. Ellis, and C.J. Walsh, *Biomechanical mechanisms underlying exosuit-induced improvements in walking economy after stroke.* Journal of Experimental Biology, 2018. **221**(5): p. jeb168815. DOI: 10.1242/jeb.168815.
123. Russell Esposito, E.S., Kelly A.: Wilken, Jason M., *Experimental comparisons of passive and powered ankle-foot orthoses in individuals with limb reconstruction.* Journal of NeuroEngineering and Rehabilitation, 2018. **15**(1). DOI: 10.1186/s12984-018-0455-y.
124. Roy, A.K., Hermano I.: Patterson, Shawnna L.: Judkins, Timothy N.: Khanna, Ira: Forrester, Larry W.: Macko, Richard M.: Hogan, Neville. *Measurement of Human Ankle Stiffness Using the Anklebot*. 2007. IEEE.DOI: 10.1109/icorr.2007.4428450.
125. Roy, A.K., H. I.: Williams, D. J.: Bever, C. T.: Forrester, L. W.: Macko, R. M.: Hogan, N., *Robot-Aided Neurorehabilitation: A Novel Robot for Ankle Rehabilitation.* IEEE Transactions on Robotics, 2009. **25**(3): p. 569-582. DOI: 10.1109/tro.2009.2019783.

126. Roy, A.K., Hermano I.: Barton, Joseph E.: Macko, Richard F.: Forrester, Larry W. *Anklebot-assisted locomotor training after stroke: A novel deficit-adjusted control approach*. 2013. IEEE.DOI: 10.1109/icra.2013.6630869.
127. Roy, A.K., Hermano I.: Iqbal, Kamran: Macko, Nathan R.: Macko, Richard F.: Forrester, Larry W. *Facilitating push-off propulsion: A biomechanical model of ankle robotics assistance for plantarflexion gait training in stroke*. 2014. IEEE.DOI: 10.1109/biorob.2014.6913853.
128. Roy, A.C., Christopher: Forrester, Larry W.: Hafer-Macko, Charlene E.: Macko, Richard F. *Quantifying Human Autonomy Recovery During Ankle Robot-Assisted Reversal of Foot Drop After Stroke*. 2018. IEEE.DOI: 10.1109/biorob.2018.8487221.
129. Noda, T.T., Asuka: Teramae, Tatsuva: Hirookai, Eiko: Hase, Kimitaka: Morimoto, Jun. *Robotizing Double-Bar Ankle-Foot Orthosis*. 2018. IEEE.DOI: 10.1109/icra.2018.8462911.
130. Koller, J.R.G., D. H.: Ferris, D. P.: Remy, C. D., *Confidence in the curve: Establishing instantaneous cost mapping techniques using bilateral ankle exoskeletons.* J Appl Physiol (1985), 2017. **122**(2): p. 242-252. DOI: 10.1152/japplphysiol.00710.2016.
131. Koller, J.R.D.R., C.: Ferris, Daniel P. *Comparing neural control and mechanically intrinsic control of powered ankle exoskeletons*. 2017. IEEE.DOI: 10.1109/icorr.2017.8009262.
132. Koller, J.R.R., C. David: Ferris, Daniel P., *Biomechanics and energetics of walking in powered ankle exoskeletons using myoelectric control versus mechanically intrinsic control.* Journal of NeuroEngineering and Rehabilitation, 2018. **15**(1). DOI: 10.1186/s12984-018-0379-6.
133. Jacobs, D.A.K., Jeffrey R.: Steele, Katherine M.: Ferris, Daniel P., *Motor modules during adaptation to walking in a powered ankle exoskeleton.* Journal of NeuroEngineering and Rehabilitation, 2018. **15**(1). DOI: 10.1186/s12984-017-0343-x.
134. Durandau, G., W.F. Rampeltshammer, H. Van Der Kooij, and M. Sartori. *Myoelectric model-based control of a bi-lateral robotic ankle exoskeleton during even ground locomotion*. in *2020 8th IEEE RAS/EMBS International Conference for Biomedical Robotics and Biomechatronics (BioRob)*. 2020. IEEE.
135. Durandau, G., W. Rampeltshammer, H. van der Kooij, and M.J.a.p.a. Sartori, *Neuromechanical model-based control of bi-lateral ankle exoskeletons: biological joint torque and electromyogram reduction across walking conditions.* 2021.
136. Shorter, K.A., G.F. Kogler, E. Loth, W.K. Durfee, and E.T. Hsiao-Wecksler, *A portable powered ankle-foot orthosis for rehabilitation.* Journal of Rehabilitation Research & Development, 2011. **48**(4).
137. Li, D.Y., A. Becker, K.A. Shorter, T. Bretl, and E.T.J.I.A.T.o.M. Hsiao-Wecksler, *Estimating system state during human walking with a powered ankle-foot orthosis.* 2011. **16**(5): p. 835-844.
138. Shorter, K.A., Y. Li, E.A. Morris, G.F. Kogler, and E.T. Hsiao-Wecksler. *Experimental evaluation of a portable powered ankle-foot orthosis*. 2011. IEEE.DOI: 10.1109/iembs.2011.6090138.

139. Petrucci, M.N.M., Colum D.: Hsiao-Wecksler, Elizabeth T. *Modulation of anticipatory postural adjustments of gait using a portable powered ankle-foot orthosis*. 2013. IEEE.DOI: 10.1109/icorr.2013.6650450.
140. Li, Y.D.H.-W., Elizabeth T. *Gait mode recognition and control for a portable-powered ankle-foot orthosis*. 2013. IEEE.DOI: 10.1109/icorr.2013.6650373.
141. Boes, M.K.I., Mazharul: David Li, Yifan: Hsiao-Wecksler, Elizabeth T. *Fuel efficiency of a Portable Powered Ankle-Foot Orthosis*. 2013. IEEE.DOI: 10.1109/icorr.2013.6650445.
142. Boes, M.K.B., Rachel E.: Kesler, Richard M.: Learmonth, Yvonne C.: Islam, Mazharul: Petrucci, Matthew N.: Motl, Robert W.: Hsiao-Wecksler, Elizabeth T., *Six-Minute Walk Test Performance in Persons With Multiple Sclerosis While Using Passive or Powered Ankle-Foot Orthoses.* Archives of Physical Medicine and Rehabilitation, 2018. **99**(3): p. 484-490. DOI: 10.1016/j.apmr.2017.06.024.
143. Baser, O.K., Hasbi, *Mechanical design and preliminary tests of VS-AnkleExo.* Journal of the Brazilian Society of Mechanical Sciences and Engineering, 2018. **40**(9). DOI: 10.1007/s40430-018-1365-4.
144. Zhu, J.S., Haotian: Sun, Wentao: Li, Xin: Zhang, Xiaohua: Huang, Qiang. *Design of compliant joints and human-robot connection in an ankle-foot exoskeleton*. 2017. IEEE.DOI: 10.1109/cbs.2017.8266077.
145. Zhu, J., Q. Wang, Y. Huang, and L. Wang. *Adding compliant joints and segmented foot to bio-inspired below-knee exoskeleton*. in *2011 IEEE International Conference on Robotics and Automation*. 2011. IEEE.
146. Xu, K.L., Juan: Li, Weida: Cai, Xiaowei: Xia, Ziyu. *A hybrid powered ankle exoskeleton for walking*. 2017. IEEE.DOI: 10.1109/cac.2017.8243433.
147. Chinh, N.D.T., Pham Nhat: Long, Chu Ba: Tien, Nguyen Tan. *Design of force support device for human ankle joint*. 2017. IEEE.DOI: 10.1109/urai.2017.7992728.
148. Noël, M., B. Cantin, S. Lambert, C.M. Gosselin, L.J.J.I.T.o.N.S. Bouyer, and R. Engineering, *An electrohydraulic actuated ankle foot orthosis to generate force fields and to test proprioceptive reflexes during human walking.* 2008. **16**(4): p. 390-399.
149. Noel, M.F., Karine: Bouyer, Laurent J., *Using an electrohydraulic ankle foot orthosis to study modifications in feedforward control during locomotor adaptation to force fields applied in stance.* Journal of NeuroEngineering and Rehabilitation, 2009. **6**(1): p. 16. DOI: 10.1186/1743-0003-6-16.
150. Fournier Belley, A.B., Jason: Brochu, Karine: Mercier, Catherine: Roy, Jean-Sébastien: Bouyer, Laurent, *Development and reliability of a measure evaluating dynamic proprioception during walking with a robotized ankle-foot orthosis, and its relation to dynamic postural control.* Gait & Posture, 2016. **49**: p. 213-218. DOI: 10.1016/j.gaitpost.2016.07.013.
151. Gu, G.M.K., Seulki: Park, Dae-Sung: Kim, Jung. *SMAFO: Stiffness modulated Ankle Foot Orthosis for a patient with foot drop*. 2015. IEEE.DOI: 10.1109/icorr.2015.7281256.

152. Dhule, S.V.S., V. V.: Somani, S. B. *Implementation of adaptive dorsiflexion and plantar flexion in active ankle foot orthosis*. 2015. IEEE.DOI: 10.1109/infop.2015.7489475.
153. Park, Y.-L.C., Bor-Rong: Young, Diana: Stirling, Leia: Wood, Robert J.: Goldfield, Eugene: Nagpal, Radhika. *Bio-inspired active soft orthotic device for ankle foot pathologies*. 2011. IEEE.DOI: 10.1109/iros.2011.6094933.
154. Park, Y.-L.C., Bor-rong: Pérez-Arancibia, Néstor O: Young, Diana: Stirling, Leia: Wood, Robert J: Goldfield, Eugene C: Nagpal, Radhika, *Design and control of a bio-inspired soft wearable robotic device for ankle–foot rehabilitation.* Bioinspiration & biomimetics, 2014. **9**(1): p. 016007.
155. Murphy, P.A., Garrick: Daly, Sean: Bolton, Michael: Maurice, Oliver: Bonia, Thomas: Mavroidis, Constantinos: Sheng-Che, Yen. *Test of a customized compliant ankle rehabilitation device in unpowered mode*. 2014. IEEE.DOI: 10.1109/embc.2014.6944268.
156. Rahman, S.M.M.I., Ryojun, *A Novel Variable Impedance Compact Compliant Ankle Robot for Overground Gait Rehabilitation and Assistance.* Procedia Engineering, 2012. **41**: p. 522-531. DOI: 10.1016/j.proeng.2012.07.207.
157. Polinkovsky, A.B., Richard J.: Kern, Nicole I.: Quinn, Roger D. *An Ankle Foot Orthosis with Insertion Point Eccentricity Control*. 2012. IEEE.DOI: 10.1109/iros.2012.6386193.
158. Patar, A.J., Norman: Makhtar, Khushairy: Mahmud, Jamaluddin: Komeda, Takashi, *Development of Dynamic Ankle Foot Orthosis for Therapeutic Application.* Procedia Engineering, 2012. **41**: p. 1432-1440. DOI: 10.1016/j.proeng.2012.07.332.
159. Hamid, A.P., Mohd Nor Azmi Ab: Ayub, Muhammad Azmi, *Force Sensor Detection and Performance Evaluation of New Active System Ankle Foot Orthosis.* Procedia Engineering, 2012. **41**: p. 510-515. DOI: https://doi.org/10.1016/j.proeng.2012.07.205.
160. Bharadwaj, K.S., Thomas G: Koeneman, James B: Koeneman, Edward J, *Design of a robotic gait trainer using spring over muscle actuators for ankle stroke rehabilitation.* 2005.
161. Ward, J.A., S. Balasubramanian, T. Sugar, and J. He. *Robotic gait trainer reliability and stroke patient case study*. in *2007 IEEE 10th International Conference on Rehabilitation Robotics*. 2007. IEEE.
162. Oymagil, A.M.H., Joseph K.: Sugar, Thomas: Fleeger, Jennifer. *Control of a Regenerative Braking Powered Ankle Foot Orthosis*. 2007. IEEE.DOI: 10.1109/icorr.2007.4428402.
163. Hitt, J.O., A. Mehmet: Sugar, Thomas: Hollander, Kevin: Boehler, Alex: Fleeger, Jennifer. *Dynamically Controlled Ankle-Foot Orthosis (DCO) with Regenerative Kinetics: Incrementally Attaining User Portability*. 2007. IEEE.DOI: 10.1109/robot.2007.363543.
164. Boehler, A.W.H., Kevin W.: Sugar, Thomas G.: Dosun, Shin. *Design, implementation and test results of a robust control method for a powered ankle foot orthosis (AFO)*. 2008. IEEE.DOI: 10.1109/robot.2008.4543504.
165. Ward, J.S., Thomas: Standeven, John: Engsberg, Jack R. *Stroke survivor gait adaptation and performance after training on a Powered Ankle Foot Orthosis*. 2010. IEEE.DOI: 10.1109/robot.2010.5509957.

166. Ward, J.S., Thomas: Boehler, Alexander: Standeven, John: Engsberg, Jack R., *Stroke Survivors' Gait Adaptations to a Powered Ankle–Foot Orthosis.* Advanced Robotics, 2011. **25**(15): p. 1879-1901. DOI: 10.1163/016918611x588907.
167. Caltran, C.S., Adriano A. G.: Inoue, Roberto S.: Terra, Marco H., *Robust Filtering Applied to Position Estimation of an Active Ankle-Foot Orthosis.* IFAC Proceedings Volumes, 2011. **44**(1): p. 9391-9396. DOI: 10.3182/20110828-6-it-1002.03598.
168. Jardim, B.S., Adriano A. G., *Development and analysis of series elastic actuators for impedance control of an active ankle–foot orthosis.* Journal of the Brazilian Society of Mechanical Sciences and Engineering, 2014. **36**(3): p. 501-510. DOI: 10.1007/s40430-013-0092-0.
169. Zhang, Z.W., Zhen: Jiang, Jia-Xin: Qian, Jin-Wu, *Control method for exoskeleton ankle with surface electromyography signals.* Journal of Shanghai University (English Edition), 2009. **13**(4): p. 270-273. DOI: 10.1007/s11741-009-0402-1.
170. Boiadjiev, G.V.V., Ivanka P., *Control System for Data Acquisition and Processing of Ankle-Foot Orthosis.* IFAC Proceedings Volumes, 2009. **42**(16): p. 597-602. DOI: 10.3182/20090909-4-jp-2010.00101.
171. Veneva, I. *Intelligent device for control of active ankle-foot orthosis*. in *7th IASTED international conference on biomedical engineering; 17–19 feburary*. 2010. Acta Press.
172. Norris, J.A., K.P. Granata, M.R. Mitros, E.M. Byrne, A.P.J.G. Marsh, and posture, *Effect of augmented plantarflexion power on preferred walking speed and economy in young and older adults.* 2007. **25**(4): p. 620-627.
